# Breakthrough Infection With Severe Acute Respiratory Syndrome Coronavirus 2 Among Healthcare Workers in Delhi: A Single-Institution Study

**DOI:** 10.7759/cureus.19070

**Published:** 2021-10-27

**Authors:** Pragya Sharma, Suruchi Mishra, Saurav Basu, Rajesh Kumar, Neha Tanwar

**Affiliations:** 1 Community Medicine, Maulana Azad Medical College, Delhi, IND

**Keywords:** india, covid-19 vaccination, sars-cov-2, covid-19, breakthrough infection

## Abstract

Introduction

This study aimed to determine the breakthrough infection rate of coronavirus disease 2019 (COVID-19) (severe acute respiratory syndrome coronavirus 2 {SARS-CoV-2}) infection in healthcare workers (HCWs) vaccinated with either BBV152 or AZD1222 (ChAdOx1-S) vaccine.

Methods

A cross-sectional analysis was conducted at a medical college and hospital complex in Delhi, India, through telephonic interviews among HCWs who had received at least one dose of a COVID-19 vaccine during January-March 2021. Breakthrough infections were operationally defined as the occurrence of COVID-19 infection ≥14 days after administration of two doses of either COVID-19 vaccine. Data were entered in Epidata 3.1 (Odense, Denmark: EpiData Association) (single entered) and analyzed with IBM SPSS version 25 (Armonk, NY: IBM Corp.). A p-value < 0.05 was considered statistically significant.

Results

We enrolled 325 HCWs with a mean (SD) age of 29.1 (9.9) years including 211 (64.9%) males and 114 (35.1%) females. A total of 37 (13.3%, 95% CI 9.8, 17.7) COVID-19 breakthrough infections were observed in the HCWs. Additionally, 20 (6.1%) non-breakthrough infections were reported in the HCWs who were vaccinated with at least a single dose of a COVID-19 vaccine, or both doses, but prior to 14 days since the administration of the second dose. Most breakthrough infections were mild without needing supplemental oxygen for recovery.

Conclusion

Nearly one in seven HCWs experienced a COVID-19 breakthrough infection in the present study. A history of SARS-CoV-2 natural infection followed by at least one dose of COVID-19 vaccination was associated with significant protection against breakthrough infections.

## Introduction

Vaccines are considered the mainstay in halting and ending the coronavirus disease 2019 (COVID-19) pandemic which has caused over 172 million cases and 3.7 million deaths worldwide till date [1]. India launched the world’s largest COVID-19 mass vaccination campaign from January 2021 in a phased manner beginning with healthcare, sanitation, and essential frontline workers, followed by the geriatric population, people with comorbidities, those aged > 45 years, and finally the entire adult population [2].

The vaccines approved and deployed in India by the regulatory authority included AZD1222-ChAdOx1-S (Covishield), manufactured in India by Serum Institute of India through license from AstraZeneca-Oxford [3] and BBV152 (Covaxin), the indigenous vaccine developed by Bharat Biotech in collaboration with the Indian Council of Medical Research (ICMR) [4]. The AZD122 (ChAdOx1-S/nCoV-19) recombinant vaccine against COVID-19 is a replication-deficient adenoviral vector vaccine that expresses the severe acute respiratory syndrome coronavirus 2 (SARS-CoV-2) spike protein gene [3]. BBV152 is a whole-virion inactivated SARS-CoV-2 vaccine adjuvanted with Algel-IMDG to induce T helper-1 cell (Th1) responses [4]. The efficacy of AZD1222 (ChAdOx1-S) after administration of two doses of the vaccines irrespective of the interval between the doses has been reported as 63.1%, with possibly higher efficacy on longer intervals [3]. The interim phase 3 clinical trial data reported BBV152 to have the efficacy of 78% against infection with SARS-CoV-2 [5]. However, the real-world effectiveness of vaccines may differ from the efficacy reported in clinical trials due to a multitude of factors including the dynamics of disease exposure, diminished antibody response in subgroups like the elderly and the immunocompromised, and the emergence of newer mutant strains with greater infectivity and virulence [6,7].

A small proportion of individuals will contract COVID-19 despite complete vaccination as no vaccine accords 100% protection against the disease, and occasionally newer virus variants evolve mechanisms for bypassing the vaccine-induced antibody response. Breakthrough infections with reference to COVID-19 refer to the incidence of SARS-CoV-2 infections in individuals who have already been partially or completely vaccinated with any authorized COVID-19 vaccine [8].

According to the ICMR between 0.02% and 0.04% infections have occurred after partial or complete vaccination with either BBV152 or AZD1222 (ChAdOx1-S) [9]. However, healthcare workers (HCWs) represent a very high-risk group for contracting COVID-19 due to sustained occupational exposure to the virus [10-13]. Consequently, assessment of the breakthrough infection rate in this cohort would provide crucial evidence in understanding the effectiveness of vaccination in preventing symptomatic disease and disease transmission in a highly vulnerable population. Furthermore, the estimation of non-breakthrough infections in individuals vaccinated with at least one dose of vaccine is also important from the context of lower-middle-income countries including India since the proportion of the population having received one dose is substantially larger than that having received both vaccine doses.

This study, therefore, aimed to determine the breakthrough infection rate of COVID-19 (SARS-CoV-2) infection in the healthcare workers vaccinated with either BBV152 or AZD1222 (ChAdOx1-S) vaccine.

This article was previously published as a preprint: Sharma P, Mishra S, Basu S, Tanwar N, Kumar R: Breakthrough infection with SARS-CoV-2 and its predictors among healthcare workers in a medical college and hospital complex in Delhi, India. medRxiv. 2021, pp. 1-11. DOI: 10.1101/2021.06.07.21258447.

## Materials and methods

Study design and setting

We conducted a cross-sectional study among healthcare workers (HCWs) previously vaccinated with at least one or both doses of a COVID-19 vaccine and affiliated to the Maulana Azad Medical College, New Delhi, who were mostly providing healthcare services at the largest dedicated tertiary care COVID-19 hospital in Delhi. As per the government of India policy, two doses of either BBV152 (Covaxin) or AZD1222 (Covishield) vaccine at least four weeks apart were available for administration to all HCWs since January 2021 with no mixing of doses allowed. Unvaccinated HCWs were excluded from this study. 

The primary outcome of the study was the proportion of breakthrough infection in HCWs which was defined as any COVID-19 infection occurring ≥14 days after receiving both doses of either of the vaccine(s). The secondary outcome was the proportion of non-breakthrough COVID-19 infections that occurred post-vaccination with either a single dose of a COVID-19 vaccine or both doses but prior to a period of 14 days since the administration of the second dose.

The independent variables included age, sex, time since vaccination, vaccine type, adherence to non-pharmaceutical measures post-vaccination, and previous history of natural COVID-19 infection and recovery prior to vaccination. The sample size was calculated considering the prevalence of breakthrough infection among HCWs in India as 13.3% as reported in a study from a chronic care facility in Delhi at 95% confidence level, 4% precision, and 10% non-response [14]. The minimum sample size was estimated as 270.

The data were collected during a period of seven days from May 31, 2021, to June 6, 2021, through telephonic interviews conducted by multiple trained investigators from the following sources through the consecutive sampling method: (i) registration records of all the healthcare workers vaccinated at the Covaxin administration site within the college campus during February-March 2021; (ii) records of the medical interns affiliated to the Maulana Azad Medical College (MAMC) who were vaccinated between January 2021 and March 2021. 

Statistical analysis

Data were entered in Epidata 3.1 (Odense, Denmark: EpiData Association) (single entered) and analyzed with IBM SPSS version 25 (Armonk, NY: IBM Corp.). Results were expressed in frequency and proportions for categorical variables, and mean and standard deviation for continuous variables. The significance of the difference between proportions was assessed using the chi-square test. A p-value < 0.05 was considered statistically significant.

## Results

We enrolled 325 healthcare workers including 258 medical doctors and interns (79.4%), 52 (16%) frontline health workers, 12 (3.7%) lab technicians, and three (0.9%) nurses. The mean (SD) age of the participants was 29.1 (9.9) years including 212 (65%) males and 114 (35%) females. A total of 279 (85.8%) HCWs were fully vaccinated with two doses while 46 (14.2%) had received only one dose of a COVID-19 vaccine at the time of interview. There were 168 (51.7%) BBV152 recipients and 157 (48.3%) AZD1222 (ChAdOx1-S) recipients. Fifty (15.4%) HCWs reported a past history of natural COVID-19 infection and recovery prior to receiving the first dose of COVID-19 vaccine with 47 (94.1%) being mild cases and 3 (5.9%) being moderate cases needing supplemental oxygen therapy.

A total of 57 (17.5%, 95% confidence interval {CI} 13.8, 22.0) COVID-19 infections comprising 37 breakthrough and 20 non-breakthrough infections were observed in the HCWs vaccinated with at least one dose of COVID-19 vaccine. The infections were diagnosed with reverse transcription-polymerase chain reaction (RT-PCR), antigen (Ag) test, and on strong clinical suspicion without laboratory confirmation in 48 (84.2%), three (5.2%), and six (10.5%) cases, respectively. The severity of COVID-19 infections was mild requiring only home isolation in 51 (89.4%) cases while six (10.6%) were moderate cases needing supplemental oxygen therapy. Within the households of HCWs suffering COVID-19 infection post-vaccination with at least one dose of either COVID-19 vaccine, all members were diagnosed concurrently with COVID-19 in nine (15.8%) cases while at least one member was infected in 24 (42.1%) cases.

A total of 37 (13.3%, 95% CI 9.8, 17.7) breakthrough infections were observed in the HCWs of which 32 (86.5%), three (8.1%), and two (5.4%) cases were diagnosed with RT-PCR, Ag test, and on the basis of clinical suspicion, respectively. The median (interquartile range {IQR}) time until the incidence of COVID-19 breakthrough infection after receiving the second dose of either COVID-19 vaccine was 47 (28.5, 55) days.

Among the HCWs experiencing a breakthrough infection, 35 (94.6%) were mild cases managed through only home isolation while there were two (5.4%) moderate cases requiring supplemental oxygen therapy prior to recovery. Within the households of HCWs reporting the incidence of a COVID-19 breakthrough infection, concurrent infection in all members was observed in five (13.5%) cases while at least one member was infected in 14 (37.8%) cases.

Table 1 summarizes the COVID-19 infection rates in HCWs after administration of at least a single dose of a COVID-19 vaccine. HCWs without a history of natural COVID-19 infection and recovery prior to vaccination compared to HCWs with such a history were having 3.8 times (95% CI OR 1.1, 12.7) higher risk of contracting a COVID-19 infection or reinfection despite vaccination with at least one dose of either COVID-19 vaccine (p=0.029). 

**Table 1:** Distribution of COVID-19 infections post first dose of vaccination in HCWs (N=325) *Thirty-seven breakthrough and 20 non-breakthrough infections. **Prior to administration of the first dose of COVID-19 vaccine. ***Post-vaccination with at least one dose of vaccine. COVID-19: coronavirus disease 2019; HCWs: healthcare workers

Characteristic	Total (N=325)	Total infections^*^(n=57)	Unadjusted odds 95% CI	p-Value
Vaccination status				
Two doses	279 (85.8%)	45 (16.1%)	1	0.104
One dose	46 (14.2%)	12 (26.1%)	0.54(0.26,1.1)	
Vaccine type				
BBV152	168 (51.7%)	33 (19.6%)	0.74 (0.41, 1.3)	0.303
AZD1222	157 (48.3%)	24 (15.3%)	1	
Age (years)				
<35	255 (78.5%)	46 (18%)	1	0.651
≥35	70 (21.5%)	11 (15.7%)	1.2 (0.57, 2.4)	
Sex				
Male	211 (64.9%)	42 (19.9%)	1	0.129
Female	114 (35.1%)	15 (13.2%)	1.6 (0.86, 3.1)	
HCW type				
Doctor	258 (79.4%)	48 (18.6%)	1	0.324
Other	67(20.6%)	9 (13.4%)	1.4 (0.68, 3.2)	
History of COVID-19 infection^**^				
Present	50 (15.4%)	3 (6.0%)	3.8 (1.1, 12.7)	0.029
Absent	275 (84.6%)	54 (19.6%)	1	
Masking adherence^***^				
Always	225 (69.2%)	43 (19.1%)	1	0.265
Mostly/other	100 (30.8%)	14 (14%)	1.4 (0.75, 2.8)	
Social distancing adherence^***^				
Always	206 (63.4%)	34 (16.5%)	1	0.520
Mostly/other	119 (36.6%)	23 (19.3%)	0.82 (0.46, 1.5)	

The proportion of breakthrough infections did not show statistically significant variation when compared across subgroups including the type of vaccine, age, sex, adherence to non-pharmaceutical measures after vaccination, and history of a natural COVID-19 infection prior to vaccination (Table 2).

**Table 2:** Distribution of factors associated with COVID-19 breakthrough infections in HCWs (N=279)*** *Prior to administration of the first dose of COVID-19 vaccine. **Post-vaccination with at least one dose of vaccine. ***Excluding those HCWs who received only a dose of vaccine (n=46). COVID-19: coronavirus disease 2019; HCWs: healthcare workers

Characteristic	Total (N=279)	Breakthrough infections (n=37)	Unadjusted odds 95% CI	p-Value
Vaccine type				
BBV152	154 (55.2%)	24 (15.6%)	1.5 (0.77, 3.3)	0.219
AZD1222	125 (44.8%)	13 (10.4%)	1	
Age (years)				
<35	215 (77.1%)	31 (14.4%)	1	0.401
≥35	64 (22.9%)	6 (9.4%)	0.61 (0.24, 1.5)	
Sex				
Male	178 (63.8%)	27 (15.2%)	1	0.271
Female	101 (36.2%)	10 (9.9%)	1.6 (0.7, 3.5)	
HCW type				
Doctor	217 (77.8%)	32 (14.7%)	1	0.206
Other	62 (22.2%)	5 (8.1%)	0.5 (0.19, 1.4)	
History of COVID-19 infection^*^				
Present	40 (14.3%)	3 (7.5%)	0.49 (0.14, 1.7)	0.319
Absent	239 (85.7%)	34 (14.2%)	1	
Masking adherence^**^				
Always	190 (68.1%)	26 (13.7%)	1	0.851
Mostly/other	89 (31.9%)	11 (12.4%)	0.89 (0.42, 1.9)	
Social distancing adherence^**^				
Always	172 (61.6%)	21 (12.2%)	1	0.315
Mostly/other	107 (38.4%)	16 (15.0%)	1.3 (0.63, 2.5)	

## Discussion

In this study, nearly one in five HCWs reported the incidence of COVID-19 infection after receiving at least one dose of a COVID-19 vaccine. Furthermore, nearly one in seven HCWs experienced a breakthrough infection after being administered both scheduled doses of the COVID-19 vaccine. These findings suggest that in real-world settings a significant proportion of vaccinated individuals with a high risk of exposure remain vulnerable to COVID-19 infection albeit with reduced disease severity in most cases. 

Vaccine seroconversion through robust anti-spike antibody response is likely to be induced after a single dose of AZD1222 (ChAdOx1-S) compared to BBV152 wherein two doses are usually required to stimulate adequate antibody levels [7]. However, in the present study, although breakthrough and non-breakthrough infections were higher in the BBV152 group compared to the AZD1222 group, the differences were not statistically significant.

Evidence from a previous study suggests that a single dose of either BBV152 or AZD1222 induced a higher concentration of neutralizing IgG antibodies in those having a history of natural infection and recovery from COVID-19 [15]. Similarly, in this study, a history of natural infection and recovery from COVID-19 was observed to be protective against subsequent COVID-19 infection or reinfection in those HCWs who had been administered at least a single dose of COVID-19 vaccine.

The rates of breakthrough infection observed in the present study (13.3%) were similar to that observed during surveillance in a chronic care facility in Delhi, India, (13.2%) where HCWs received either AZD1222 (ChAdOx1-S) or BBV152 [14]. However, another study in a large cohort of HCWs from a north Indian city vaccinated with AZD1222 (ChAdOx1-S) reported the incidence of COVID-19 breakthrough infections to be only 1.6% (48 out of 3000) while 2.6% tested positive after receiving at least one dose of the vaccine [16]. In contrast, Hacisuleyman et al. report the incidence of breakthrough infection as just 0.5% in a cohort of 417 health care workers who had previously received two doses of BNT162b2 (Pfizer-BioNTech) or mRNA-1273 (Moderna) vaccine [17]. 

The period of observation in this study coincided with a massive wave of the COVID-19 epidemic in Delhi during April 2021 and May 2021 which witnessed 0.737 million cases including 11,075 deaths [18]. Furthermore, the emergent evidence from genomic analysis also reveals that the COVID-19 variants of concern, B.1.617.2 (Delta) and B.1.1.7 (Alpha) having ~50% higher transmissibility were primarily responsible for the surge in cases during the same period. These variants also constituted the predominant lineages found in the breakthrough infections cases due to a probable immune escape mechanism that could occasionally bypass the vaccine-induced immunity [19]. Moreover, diminished neutralizing antibody activity and limited protectiveness against the delta variant of the SARS-CoV-2 has been observed in most of the currently available COVID-19 vaccines globally especially prior to complete vaccination with both vaccine doses [20,21].

The strengths of the study are that it was conducted in real-world settings with the period of observation inclusive of the peak of the COVID-19 pandemic in Delhi, India, when health systems were overwhelmed resulting in large-scale viral exposure of HCWs providing either outpatient or inpatient treatment services. However, there are certain study limitations. First, since the infection status of the HCWs was based on self-report in the absence of a mandatory testing policy, mostly symptomatic breakthrough infections diagnosed with RT-PCR or the Ag test were likely to be captured while asymptomatic infections also capable of viral transmission were potentially omitted. Second, although we observed high rates of transmission in the household members of the HCWs, the vaccination status in the infected household members was not recorded. Third, comorbidity status was not ascertained in the participants but the likelihood of underlying morbidities in the HCWs was less considering their low median age. Fourth, six COVID-19 infections were recognized through clinical correlation without laboratory confirmation as false positive RT-PCR cases could not be ruled out during the peak of the pandemic. Consequently, future studies should assess the extent of vaccine-induced antibody response and protection after vaccination with COVID-19 vaccines in patients with diabetes, heart disease, chronic kidney disease, and older people at risk of reduced protection in addition to pre-existing concerns causally linked with the occurrence of severe disease. A final limitation of this study is that the sample size was not adequately powered to detect statistically significant differences between subgroups.

## Conclusions

In conclusion, breakthrough infections were observed in 13.3% of completely vaccinated healthcare workers in a dedicated COVID-19 tertiary care hospital in Delhi, India, mostly during the period of the second wave of the COVID-19 pandemic in Delhi, India. None of the sociodemographic or the reported adherence to non-pharmaceutical interventions were associated with the incidence of breakthrough infections although a history of SARS-CoV-2 infection prior to vaccination could accentuate the protection. 

Consequently, breakthrough infections represent a major public health challenge in ending the COVID-19 pandemic. Robust surveillance through large-scale epidemiological studies to identify the predictors of breakthrough infection among individuals at risk, and rapid genomic analysis for early recognition of emerging variants of concern that have a greater capability of causing breakthrough infections warrant continued prioritization.
